# PW03-020 – A decade of ANTI-IL-1 therpay for CAPS in the UK

**DOI:** 10.1186/1546-0096-11-S1-A246

**Published:** 2013-11-08

**Authors:** T Lane, NM Stewart, A Bybee, DM Rowczenio, K Wynne, H Trojer, A Baginska, PA Brogan, PN Hawkins, HJ Lachmann

**Affiliations:** 1 National Amyloidosis Centre, University College London, UK; 2 Rheumatology and Vasculitis, Great Ormond Strret Hospital NHS Trust, London, UK

## Introduction

In October 2002, the first patient with CAPS was treated successfully with the anti-IL-1 agent anakinra at our Centre in the UK, and in 2009 a nationally funded canakinumab treatment service initiated for CAPS in England. By the end of 2012, 82 symptomatic individuals have been assessed at our Centre.

## Objectives

To describe our experience, and outcomes of 82 individuals with clinical CAPS, including treatment and natural history.

## Methods

We examined all available medical and laboratory records.

## Results

Of 82 patients with clinical CAPS a pathogenic sequence variant (PSV) was detected in 77 (94%); 17 PSVs were identified, the commonest were R260W (27%), A439V (23%), T348M (17%), V198M (5%). 5 patients had no PSV detected on Sanger sequencing: 3 children with CINCA and 2 patients with adult onset clinical MWS. 90% were white, 8.5% South Asian and 1% African. 46 (56%) gave a positive family history. There were 8 CINCA cases, 4 MWS/CINCA overlap (75% T348M), 59 MWS (62% A439V, 31% R260W, 19% T348M, 5% V198M) and 11 FCAS (36% A439V, 27% R260W, 9% V198M). Hearing impairment was present in 31 (38%); 12/14 patients (86%) with T348M, 1/19 (5%) of A439V, 3/22 (14%) of R260W and 3/4 (75%) of V198M.

51 patients are receiving canakinumab (20 having converted from anakinra). Over a median follow up (FU) period of 28 months (IQR 15-40), 43 (84%) have experienced complete remission (CR) of disease activity whilst the remainder (8, 16%) have experienced partial remission (PR), defined as good but incomplete resolution of symptoms or serum inflammatory markers. 15 (29%) patients are being treated with double the licensed dose – 4 CINCA (including 2 children), 4 MWS/CINCA overlap, 6MWS (including 2 children) and 1 FCAS with uveitis. 3 patients have discontinued canakinumab and are currently on no treatment (one each with A439V, T348M, R260W) due to: an episode of diverticulitis; pregnancy; a desire for a treatment break. Serious adverse events included infections (diverticulitis, UTI, tonsillitis).

24 patients are on anakinra and over a median FU of 47 months (IQR 12-72) 20 remain in CR and 4 in PR. 6 patients have previously tried canakinumab; 2 had CR but were converted due to: planned pregnancy; planned insertion of a ventricular peritoneal shunt. One adult CINCA patient with a good PR could not tolerate travel to our Centre. A female with mutation negative MWS previously in CR on anakinra opted for a trial of canakinumab, but developed a massive disease flare resulting in hospitalisation. She subsequently reverted to anakinra and is once again in CR. 2 males (A439V, T346I) discontinued canakinumab due to: lack of efficacy; development of major systemic inflammation and a morphea like rash. On anakinra the former remains in PR whilst the latter has experienced CR.

2 children with mild R260W disease have declined treatment.

## Conclusion

In this series T348M underlies more severe disease than the other common mutations. A decade of IL-1 blockade confirms its efficacy and relative safety in CAPS across the clinical severity spectrum. Canakinumab is the more popular drug due to its long action; however, a small number of patients are unresponsive to this therapy, suggesting a possible role of IL-1α.

## Disclosure of interest

None declared

